# Laboratory management of Crimean-Congo haemorrhagic fever virus infections: perspectives from two European networks

**DOI:** 10.2807/1560-7917.ES.2019.24.5.1800093

**Published:** 2019-01-31

**Authors:** Barbara Bartolini, Cesare EM Gruber, Marion Koopmans, Tatjana Avšič, Sylvia Bino, Iva Christova, Roland Grunow, Roger Hewson, Gulay Korukluoglu, Cinthia Menel Lemos, Ali Mirazimi, Anna Papa, Maria Paz Sanchez-Seco, Aisha V. Sauer, Hervè Zeller, Carla Nisii, Maria Rosaria Capobianchi, Giuseppe Ippolito, Chantal B. Reusken, Antonino Di Caro

**Affiliations:** 1 ‘L. Spallanzani’ National Institute for Infectious Diseases IRCCS (INMI), WHO Collaborating Center for Clinical Care, Diagnosis, Response and Training on Highly Infectious Diseases, Rome, Italy; 2 Erasmus MC, Department of Viroscience, WHO Collaborating Centre for Arbovirus and Viral Hemorrhagic Fever Reference and Research, Rotterdam, The Netherlands; 3 Institute of Microbiology and Immunology, Faculty of Medicine, Ljubljana, Slovenia; 4 Control of Infectious Diseases Department Institute of Public Health, Tirana, Albania; 5 National Center of Infectious and Parasitic Diseases, Sofia, Bulgaria; 6 Robert Koch Institute, Berlin, Germany; 7 Public Health England, National Infection Service WHO Collaborating Centre for Virus Reference and Research (Special Pathogens), Porton Down, Salisbury, United Kingdom; 8 Public Health General Directorate of Turkey, AnkaraCity, Turkey; 9 Consumers, Health, Agriculture and Food Executive Agency (CHAFEA), Luxembourg, Luxembourg; 10 Public Health agency of Sweden, Solna, Sweden; 11 National Veterinary Institute, Uppsala, Sweden; 12 Department of Laboratory Medicine, Clinical Microbiology, Karolinska Institute and Karolinska University Hospital, Solna, Sweden; 13 Department of Microbiology, Medical School, Aristotle University of Thessaloniki, Thessaloniki, Greece; 14 National Centre of Microbiology, Institute of Health Carlos III, Madrid, Spain; 15 European Commission, Directorate General for Health and Food Safety, Unit for Crisis Management and Preparedness in Health, Luxembourg, Luxembourg; 16 European Center for Disease Prevention and Control, Office of the Chief Scientist, Stockholm, Sweden; 17 Centre for Infectious Disease Control, National Institute for Public Health and the Environment (RIVM), Bilthoven, the Netherlands; 18 Authors contributed equally to the work and share last authorship

**Keywords:** CCHF, Crimean-Congo haemorrhagic fever virus, CCHFV, laboratory preparedness, laboratory response, emerging diseases, European network

## Abstract

**Background:**

Crimean-Congo haemorrhagic fever virus (CCHFV) is considered an emerging infectious disease threat in the European Union. Since 2000, the incidence and geographic range of confirmed CCHF cases have markedly increased, following changes in the distribution of its main vector, *Hyalomma* ticks.

**Aims:**

To review scientific literature and collect experts’ opinion to analyse relevant aspects of the laboratory management of human CCHF cases and any exposed contacts, as well as identify areas for advancement of international collaborative preparedness and laboratory response plans.

**Methods:**

We conducted a literature review on CCHF molecular diagnostics through an online search. Further, we obtained expert opinions on the key laboratory aspects of CCHF diagnosis. Consulted experts were members of two European projects, EMERGE (Efficient response to highly dangerous and emerging pathogens at EU level) and EVD-LabNet (Emerging Viral Diseases-Expert Laboratory Network).

**Results:**

Consensus was reached on relevant and controversial aspects of CCHF disease with implications for laboratory management of human CCHF cases, including biosafety, diagnostic algorithm and advice to improve lab capabilities. Knowledge on the diffusion of CCHF can be obtained by promoting syndromic approach to infectious diseases diagnosis and by including CCHFV infection in the diagnostic algorithm of severe fevers of unknown origin.

**Conclusion:**

No effective vaccine and/or therapeutics are available at present so outbreak response relies on rapid identification and appropriate infection control measures. Frontline hospitals and reference laboratories have a crucial role in the response to a CCHF outbreak, which should integrate laboratory, clinical and public health responses.

## Introduction

Crimean-Congo haemorrhagic fever virus (CCHFV) is a tick-borne pathogen that causes a frequently lethal disease in humans and is considered to be a major emerging infectious disease threat spreading to and within Europe [1-3].

The severity of the disease, the presence of domestic and wild animal reservoirs and/or vectors, a large population of susceptible humans, limited diagnostic capacities and resources for epidemiological/ecological investigation, as well as the absence of effective prophylaxis and treatment render CCHFV a pathogen with outbreak potential [4].

Since 2000, the incidence and geographic range of CCHF cases have markedly increased [5,6] following an expanding distribution of its main vector, ticks of the genus *Hyalomma*, specifically the *Hyalomma marginatum* species [1,7,8]. In Turkey, nearly 900 new CCHF cases occur annually, with a total of 9,787 cases reported from 2002–15 [9]. CCHF is endemic in the Balkan region, in Kosovo, 228 cases were reported from 1995–2013 [10], In Bulgaria, over 1,500 cases have been reported from 1952 [11]. In the European region, cases of human infection have also been reported from Albania, Russian Federation, Georgia, Greece, and Ukraine [12]. Imported cases have been reported in France [13], United Kingdom [14], Greece [15] and Germany [16]. A detailed review of other outbreaks has been recently published by Papa et al. [11].

Public health systems (including diagnostic laboratories) should be prepared to respond to the increased circulation of the virus in endemic EU countries, the potential for importation of human CCHF cases or the emergence of virus circulation in new areas e.g. Spain [17].

The objectives of this study were to amalgamate the expertise of two EU expert networks (i) EMERGE (Efficient response to highly dangerous and emerging pathogens at EU level) [18] and (ii) EVD-LabNet (Emerging Viral Diseases Laboratory Network) [19], in order to select and analyse the relevant and some of controversial aspects of CCHF disease diagnostics with implications for laboratory management of human CCHF cases and any exposed contacts.

## Methods

We carried out an on line research of published paper related to CCHFV molecular detection methods. References were obtained by an online search in PubMed using an intentionally wide search-query to ensure that a large number of papers was retrieved also for a rare disease such as CCHF.

The query produced a large number of papers, 20% of them were discarded after a narrative review, as they did not contain a detailed description of the detection methods employed including the nucleotide sequences of primers and/or probes. The search was done by one author and the results discussed among the authors. Papers related on non-previously retrieved molecular detection methods or to others relevant aspects discussed in this report have been directly provided by experts. For phylogenetic analysis all available CCHF virus genomes by 5 December 2017 were retrieved from GenBank (https://www.ncbi.nlm.nih.gov/nucleotide), using ‘txid1980519(Organism)’ as term of query. All analyses have been focused only on CCHFV S-segment, because it resulted as the most conserved gene across CCHFVs [8,20] and also because mostly all retrieved molecular methods has S segment as target. CCHF virus strains with complete S segment were selected and clustered at 100% with CD-HIT v4.6. A total of 163 sequences available at 5 December 2017 were obtained and aligned with MAFFT v7.123b in local pair mode. Phylogenetic analysis were performed with RAxML v8.2.10 using GTRGAMMA model and 1000 bootstrap inferences.

A preliminary text was drafted and discussed among the experts by email and during EMERGE and EVD-LabNet networks’ 2017 and 2018 annual meetings. Most of the relevant and some of controversial aspects of CCHF disease with implications for laboratory management have been selected and analysed in the following sections. In the present paper, all the expressed opinions take into account both published data and personal experience of the experts.

## Results

### Crimean-Congo haemorrhagic fever virus clades distribution

CCHFV (family *Nairoviridae*, genus *Orthonairovirus*) is tick-borne and is maintained in a tick-vertebrate-tick cycle with *Hyalomma marginatum*, the main vector species in Europe. Given the wide distribution of its vector, CCHFV has been detected over a wide geographic range: Africa, Europe, Asia and the Middle East [5,21].

CCHFV is an enveloped, tri-partite, negative-sense, RNA virus. The large genome segment (L) encodes the RNA-dependent RNA polymerase (L protein), the medium segment (M) encodes the glycoproteins GN and GC, while the small segment (S) encodes the nucleocapsid protein (N).

**Figure 1:**
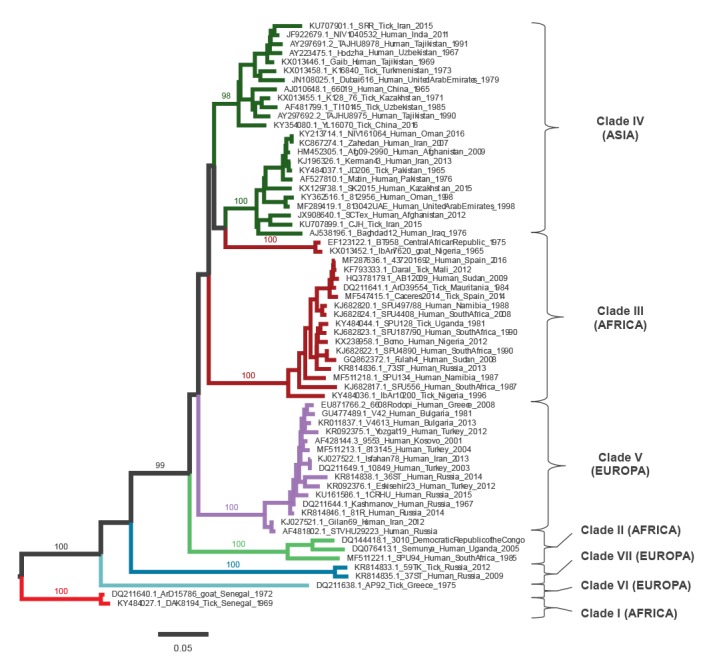
Maximum likelihood phylogenetic analysis for complete S segment of Crimean-Congo haemorrhagic fever virus (n = 65)

 Phylogenetic tree (Figure 1) was built, including only 65 of 163 representative strains with reported location of provenance either in GenBank records or in the associated papers. Taking into account similarity and geographic locations of the different viral lineages, seven genetic clades were identified: three prevalently diffused in Africa (clades I-III), three in Europe (clades V, VI and VII) and one in the south of Asia (clade IV).

Crimean-Congo haemorrhagic fever virus strains with complete S segment available as at 5 December 2017, were collected from GenBank database and clustered at 100% with CD-HIT v4.6. Sequences were aligned with MAFFT v7.123b. Phylogenetic analysis were performed using RAxML v8.2.10 with GTRGAMMA model and 1,000 bootstrap inferences. For graphical exemplification, only representative sequences in each clade for each country were selected and reported in the phylogenetic tree.

Branches owing to different clades are presented in the following colours: Africa: clade I (red), clade II (light green) and clade III (brown); Asia: clade IV (dark green); Europe: clade V (purple), clade VI (light blue) and clade VII (blue). For each strain we reported GenBank ID, isolate ID, host, country and collection date, if present. Bootstrap values are shown for each clade.

Most of the isolates causing outbreaks in eastern Europe belong to clade V, whereas clade VI and VII include largely divergent strains isolated from ticks in Greece (including the prototype strain AP92) [21,22] and Russia (GenBank accession number KR814833 and KR814835).

Moreover, isolates belonging to the African clade III were collected from infected ticks in 2010 and 2014, and recently [23] a virus aligning to this clade was the cause of an outbreak in Spain [17].

**Figure 2:**
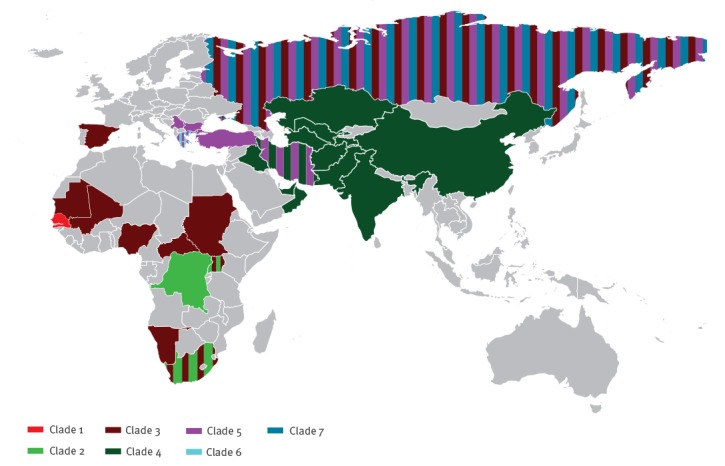
World map of Crimean-Congo haemorrhagic fever virus distribution (n = 163)

For all strains analysed, the collection country was recorded and represented on the world map in Figure 2.

Based on all available complete S segment CCHFV genomes (163 sequences as at 5 December 2017) retrieved from GenBank.

For all strains analysed, the collection countries are presented in the following colours: Africa: clade I (red), clade II (light green) and clade III (brown); Asia: clade IV (dark green); Europe: clade V (purple), clade VI (light blue) and clade VII (blue).

### Transmission mode

Human infections are usually observed as single, sporadic cases when people in rural areas are bitten by ticks that have become infected by feeding on viraemic wild and domestic animals like hares, hedgehogs, horses, livestock and possibly birds [21]. The infection in animals is generally asymptomatic; at most, a mild fever may be noted.

In addition to tick exposure, CCHF infection can result from direct contact, especially through mucous membranes or skin wounds, with crushed infected ticks or the blood of infected animals (principally among shepherds, farmers, abattoir workers and veterinarians). Person-to-person transmission can also occur through contact with virus-containing bodily fluids of patients during the first 7–10 days of illness [21]. Unprotected contact with other bodily fluids like saliva or urine, may also represent a risk for humans [24]. Nosocomial transmission to healthcare workers, transmission among patients sharing the same room [25] and possible sexual transmission [26,27] have also been reported.

### Relevance of viraemia

The typical duration of viraemia ranges from 1– 9 days [28,29], and there is so far no evidence of detectable viraemia during the incubation period [30]. However, the positivity of CCHFV RNA in serum has been exceptionally reported up to 36 days from the onset of symptoms [31]. Studies investigating the presence and persistence of CCHFV in other body fluids are limited. Viral RNA was detectable up to 10 days and as late as 25 days after onset of symptoms in saliva [24] and urine [31] respectively, but no data on virus viability are available.

Viral load is the most important prognostic factor: a value of viraemia higher than 10^8^ copies/mL is associated with fatal outcomes [32]. Viraemia decreases significantly over time in surviving patients, but remains persistently high in non-survivors [32,33].

### Antibody kinetics

All CCHFV genotypes belong to one serogroup [21]. Cross-reactivity between CCHFV and other nairoviruses infecting humans ( Erve virus [34]), has not been described, although monoclonal and polyclonal antibodies to the N protein of CCHFV were found to cross-react weakly with Dugbe virus N protein [35].

Nucleocapside-directed IgM antibodies have been identified as the initial serological marker during infection, becoming detectable in a median of 2–3 days after disease onset, followed by glycoprotein precursor (GPC) directed IgM (4–6 days) and IgG antibodies (5–6 days) [28]. In another report, CCHFV IgM was detectable from 4 days after the onset of disease for up to 4 months. The maximum level of antibody titres was usually reached in the second to third week of illness [1]. IgM titre typically declines to undetectable levels 4 months after the onset of symptoms [29].

IgG seroconversion occurs 1–2 days after the IgM response [28] and IgG antibody remains detectable for at least 5 years [29,36].

Antibody production against CCHFV is an important prognostic indicator for survival [37]. Patients with fatal outcome rarely develop measurable antibody responses (reviewed in [37]) and Saksida et al. observed a reverse correlation between viral load and antibody levels in fatal CCHF cases [33], indicating that an impaired immune response leads to uncontrolled replication of the virus. High levels of interleukin-10 (IL-10), an anti-inflammatory cytokine, were detected in patients with fatal outcomes and were lowest in patients with a moderate disease course [33]. It was hypothesised that CCHF could be the result of a delayed and downregulated immune response caused by IL-10, which leads to an increased replication and spread of CCHFV throughout the body [33].

#### Biosafety

CCHFV is classified as a risk group 4 pathogen. The virus is stable under wet conditions for 7 hours at 37 °C, 11 days at 20 °C and 15 days at 4 °C [20]. Under dry conditions, it is stable for at least 90 min, but less than 24 hours.

However, there is an ongoing debate about the absolute requirement of biosafety levels 4 (BSL4) for handling the virus [38]. Many endemic countries need to work with the virus despite the absence of BSL4 infrastructure. Biosafety and biosecurity procedures are essential for the safe and appropriate management of specimens from suspected/confirmed CCHF patients. All laboratories should refer to national guidelines on the documents ‘CWA 15793:2011 Laboratory bio-risk management’ and ‘CWA 16393:2012 Laboratory bio-risk management - Guidelines for the implementation of CWA 15793:2008’ for a complete guide [39]. The European Check List for Laboratory Biorisk Management developed in the framework of the Joint Actions Quality Assurance Exercises and Networking on the Detection of Highly Infectious Pathogens (QUANDHIP) project can be helpful for the implementation and evaluation of biorisk management approaches [40].

#### Inactivation

Like all lipid-enveloped viruses, CCHFV can be readily inactivated by common fixatives such as 2% glutaraldehyde [41], formalin and paraformaldehyde; chlorine-based disinfectants, such as 1% sodium hypochlorite [41,42]; and other disinfectants, such as hydrogen peroxide and peracetic acid [43,44]. Physical inactivation is also effective, like high temperature (56 °C for 30 min or 60 °C for 15 min) [41], Ultraviolet (1,200 to 3,000 μW/cm^2^) or low pH (less than 6) [43,45]. The virus does not survive in matured meat (due to low pH) and is also inactivated in 40% ethanol within 2 min [20].

There is a general agreement among the consulted experts that a critical aspect for laboratory biosafety and operation is the proper and reliable inactivation of specimens before they can be removed from the high-level biocontainment environment for further diagnostic testing. Some of these inactivation methods include (i) chemical treatment (i.e. Buffer AVL or Buffer RLT, Qiagen, Hilden, Germany)  + 100% ethanol, SDS, 0.5% Tween-20 (Thermofisher, Waltham, Massachusetts, USA) [46,47] or (ii) heat treatment plus riboflavin (vitamin B_2_) [48].

It is opinion of the experts that further evaluation of inactivation procedures are needed, especially for their impact on other laboratory tests necessary for clinical evaluation and increased survival rates of patients. Although there is no direct evidence of its effects on CCHFV, it has been shown that Triton X-100 (Sigma-Aldrich, Saint Louis, Missouri, USA can decrease the biohazard risk of performing laboratory tests on samples from patients infected with other haemorrhagic fever viruses (i.e. Ebola), without affecting the results of biochemical tests [49-52].

#### Transport of diagnostic samples

General guidelines for suspected viral haemorrhagic fever infections apply for the transport of diagnostic samples from CCHF-suspected cases; these are listed under guidelines as Category A, Infectious Substances Affecting Humans UN 2814 and must be transported in packaging that meets the United Nations class 6.2 specifications and complies with Packing Instruction P620 [53].

Additionally, as for other biological resources, the exchange of CCHF samples or virus strains needs to comply with the Nagoya protocol on access to genetic resources and the fair and equitable sharing of benefits arising from their utilisation, which regulates transnational exchange between countries [54].

### Diagnosis

The choice of which CCHF detection assays should be used for diagnostics with maximum sensitivity and specificity depends on the stage of disease and the specimens available.

Laboratory diagnosis of a patient with a clinical history compatible with CCHF is generally performed during the acute phase of the disease by viral RNA (RT-PCR) detection in blood [21]. In addition to blood (serum, plasma or whole blood), other possible specimens for molecular detection are saliva, urine [24,32] or post mortem biopsy of the liver and bodily fluids (including semen, for infection control purposes) [26,27].

The available data, limited to very few patients, do not allow detailed comparisons of the sensitivity of RT-PCR detection methods performed on different sample types and, in particular, of urine and saliva vs serum samples. In two of six infected patients reported in a review of CCHF cases in Kosovo* [31], viraemia was detectable up to 30 days after the onset of symptoms. In the same investigation, one patient’s urine was PCR-positive before the serum, and in another patient viruria continued longer than viraemia; however, more detailed studies on viruria are required. Further, both viruria and viraemia are detectable several days after the appearance of IgG response [31]. No chronological data are provided about detection of CCHFV RNA in saliva [24].

During the small outbreak in Spain involving two patients in 2016, positive RT-PCR results were obtained via saliva and vaginal swab, but they became negative when viraemia was still detectable [17]. Virus isolation was attempted from these samples, but was not successful (Maria Paz Sanchez-Seco, personal communication, EVD-LabNet 2^nd^ annual meeting, Rotterdam October 2017).

### Molecular detection

There is high genetic diversity within the different CCHFV strains (Figure 1), which consequently hampers the performance of molecular tests. As a result, a range of different methods employing varied primer/probe combinations have been developed and a truly universal assay has been difficult to devise. Table 1 lists published molecular assays retrieved by our PubMed search: two single round PCR, six nested PCR, 10 real-time PCR, one loop-mediated isothermal amplification (LAMP) and one recombinase polymerase amplification (RPA). Indication on reference testing materials and sensitivity/specificity of the tests are also reported, when declared.

**Table 1:** Published molecular assays for Crimean-Congo haemorrhagic fever virus detection as at 31 November 2017

Assay	Reference	Reference testing material	Declared sensitivity/specificity	Position in CCHFV strain IbAr10200	Primers and probes		
					Type	Name	Sequence
Single round PCR	Drosten 2002 [70]	Human clinical samples	95% detection limit of 2,779 copies per mL of serum	351–579	Forward primer	CCS	ATGCAGGAACCATTAARTCTTGGGA
					Reverse primer	CCAS1	CTAATCATATCTGACAACATTTC
					Additional reverse primer	CCAS2	CTAATCATGTCTGACAGCATCTC
	Deyde 2006 [71]	Human and animal laboratory isolates	ND	1–1,672	Forward primer	SF	TCTCAAAGAAACACGTGCCGC
					Reverse primer	SR	TCTCAAAGATATCGTTGCCGC
Nested PCR	Schwarz 1996 [72]	Human serum samples	ND	135–670	Forward out	F2	TGGACACCTTCACAAACTC
					Reverse out	R2	GACATCACAATTTCACCAGG
					Forward inn	F3	GAATGTGCATGGGTTAGCTC
					Reverse inn	R3	GACAAATTCCCTGCACCA
	Midili 2007 [73]	Human serum samples	ND	119–762	Forward out	CCF-115F	AARGGAAATGGACTTRTGGA
					Forward inn	CCF-131F	TGGAYACYTTCACAAACTCC
					Reverse out/inn	CCF-759R	GCAAGGCCTGTWGCRACAAGTGC
	Midili 2009 a [74]	Human serum samples	ND	170–751	Forward out	Gre-F1	AATGTGCCGAACTTGGACAG
					Reverse out	Gre-R1	TGCGACAAGTGCAATCCCG
					Forward inn	Gre-F2	ATCAGATGGCCAGTGCAACC
					Reverse inn	Gre-R2	ACTCCCTGCACCACTCAATG
	Midili 2009 b [74]	Human serum samples	ND	192–501	Forward out	Eecf-F1	TTGTGTTCCAGATGGCCAGC
					Reverse out	Eecf-R1	CTTAAGGCTGCCGTGTTTGC
					Forward inn	Eecf-F2	GAAGCAACCAARTTCTGTGC
					Reverse inn	Eecf-R2	AAACCTATGTCCTTCCTCC
	Elata 2011 [75]	Human serum samples	ND	249–700	Forward out	CCHF1	CTGCTCTGGTGGAGGCAACAA
					Reverse out	CCHF2_5	TGGGTTGAAGGCCATGATGTAT
					Forward inn	CCHFn15	AGGTTTCCGTGTCAATGCAAA
					Reverse inn	CCHFn25	TTGACAAACTCCCTGCACCAGT
	Negredo 2017 [17]	Human serum samples	ND	123–764	Forward out	CrCon1 +	RWAAYGGRCTTRTGGAYACYTTCAC
					Reverse out	CrCon1-	TRGCAAGRCCKGTWGCRACWAGWGC
					Forward inn	CriCon2 +	ARTGGAGRAARGAYATWGGYTTYCG
					Reverse inn	CriCon2-	CYTTGAYRAAYTCYCTRCACCABTC
Real-time PCR	Yapar 2005 [76]	Human serum samples	Linear detection 10^7^–10^2^ copies/mL	1,140–1,242	Forward primer	CCRealP1	TCTTYGCHGATGAYTCHTTYC
					Reverse primer	CCRealP2	GGGATKGTYCCRAAGCA
					Probe	ND	ACASRATCTAYATGCAYCCTGC
	Duh 2006 [77]	Human serum samples	Viral RNA was detected until 30 PFU/mL	296–484	Forward primer	CCHFL1	GCTTGGGTCAGCTCTACTGG
					Reverse primer	CCHFD1	TGCATTGACACGGAAACCTA
					Probe	CCHFS1	AGAAGGGGCTTGAGTGGTT
	Wolfel 2007 [40]	Human serum samples	Analytical sensitivity in concentrations ranging from 100,000 –10 copies per mL	1,068–1,248	Forward primer	RWCF	CAAGGGGTACCAAGAAAATGAAGAAGGC
					Reverse primer	RWCR	GCCACAGGGATTGTTCCAAAGCAGAC
					Probe	SE01	ATCTACATGCACCCTGCTGTGTTGACA
					Additional probe	SE03	ATTTACATGCACCCTGCCGTGCTTACA
					Additional probe	SE0A	AGCTTCTTCCCCCACTTCATTGGAGT
	Garrison 2007 [78]	Laboratory isolates	Limit of detection 10 copies/mL; from 1.18 x 10^6^ – 11.8 gene copies were linear	649–705	Forward primer	CCHF	GGAGTGGTGCAGGGAATTTG
					Reverse primer	CCHF	CAGGGCGGGTTGAAAGC
					Probe	CCHF	CAAAGGCAAGTACATCAT
	Wolfel 2009 [79]	Laboratory isolates and human serum samples	95% detection limit of 540 copies/mL of serum, corresponding to 6.3 genome copies/reaction	210–489	Forward primer	CC1a_for	GTGCCACTGATGATGCACAAAAGGATTCCATCT
					Reverse primer	CC1a_rev	GTGTTTGCATTGACACGGAAACCTATGTC
					Probe	ND	ACASRATCTAYATGCAYCCTGC
					Additional forward primer	CC1b_for	GTGCCACTGATGATGCACAAAAGGATTCTATCT
						CC1c_for	GTGCCACTGATGATGCACAAAAGGACTCCATCT
					Additional reverse primer	CC1b_rev	GTGTTTGCATTGACACGGAAGCCTATGTC
						CC1c_rev	GTGTTTGCATTGACACGGAAACCTATATC
	Atkinson 2012 [80]	Laboratory isolates	Ranging from 5x10^5^, down to 0.5 copies of S segment RNA per reaction	1–122	Forward primer	CCHFS1	TCTCAAAGAAACACGTGCC
					Reverse primer	CCHFS122	CCTTTTTGAACTCTTCAAACC
					Probe	ND	ACTCAAGGKAACACTGTGGGCGTAAG
	Jaaskelainen 2014 [81]	Laboratory isolates and human serum samples	Sensitivity 100%;specificity 97%	460–584	Forward primer	FOR	GGACATAGGTTTCCGTGTCA
					Reverse primer	REV-1	TCCTTCTAATCATGTCTGACAGC
					Additional reverse primer	REV-2	TCTGACAGCATCTCTTTGACAGAC
					Probe	probe1	TGGCGAAATTGTGATGTCTG
					Additional probe	probe2	CTTGCAGAGTACAAGGTT
					Additional probe	probe3	TRAGCAACAAAGTCCT
	Kamboj 2014 [82]	Animal	Sensitivity from 7.6 x 10^9^ –7.6 copies:specificity 100%	32–137	Forward primer	ND	AGTGTTCTCTTGAGTGCTA
					Reverse primer	ND	CCACAAGTCCATTTCCTT
					Probe	ND	ATCTCATCTTTGTTGTTCACCTC
	Pang 2014 [83]	Laboratory isolates	Limit of detection from 133 RNA copies/PCR	726–889	Forward primer	ND	GCCGTTCAGGAATAGCACTTGT
					Reverse primer	ND	TGTTATCATGCTGTCGGCRCT
					Probe	ND	CAACAGGCCTTGCYAAGCTYGCAGAGAC
Sybrgreen Real Time	Schneeberger 2017 [84]	Laboratory isolates	ND	86–244	Forward primer	ND	GATGAGATGAACAAGTGGTTTGAAGA
					Reverse primer	ND	GTAGATGGAATCCTTTTGTGCATCAT
LAMP	Osman 2013 [85]	Human serum samples	Sensitivity in detecting ≥ 0.1 fg of viral RNA 100%	1,063–1,266	Forward outer primer	F3	ACAGCCAAGAGGTACCAAGA
					Reverse outer primer	B3	GCAGCATCATCAGGGTTGG
					Inner primers	F1c	TCTGCTGAGCACCCCAAT
						F2	TGCTGGAAAGAATCGTCGGCAA
						B2	TCCCAAAGCAGACTCCCAT
						B1c	ATCTACATGCACCCTGCCGTG
					Loop primers	LF	TCATAAAGTTTCTTCCCCCACTTC
						LB	CTTACAGCAGGCAGAATCAGTG
RPA	Bonney 2017 [86]	Laboratory isolates	Limit of detection from 5x10^6^ template copies – 50 copies	8–158	Forward primer	ND	GAAACACGTGCCGCTTACGCCCACAGTGTT
					Reverse primer	ND	TAGGAGTTTGTGAAAGTGTCCATAAGTCCATT
					Probe	ND	CCGCTTACGCCCACAGTGTTCTCTTGAGTGTNTGCAAAATGGAAAACAAGATCG

CCHFV: Crimean Congo haemorrhagic fever virus; LAMP: loop-mediated isothermal amplification; ND: not declared; PFU: Plaque Forming Units; RPA: recombinase polymerase amplification; RT: reverse transcription.

For each assay, the position of amplicon is reported to respect IbAr10200 (NCBI reference sequence NC_005302). Names of each primer and probe correspond to those reported in the reference.

Therefore there is an agreement of experts that is advisable to perform more than one test to avoid exclusive reliance on a single assay and a single target, taking into account the travel history and the geographic distribution of the different strains.

### Serological assays

In published investigations, the methods employed for the detection of antibodies are indirect immunofluorescence assays (IFAs) and enzyme-linked immunosorbent assays (ELISAs) [55-57]. Several commercial kits are available (Table 2), but only the performance of Vector-Best CCHF ELISA and Euroimmun CCHF IFA have been tested in a collaborative study conducted by reference centres for CCHF laboratory diagnosis and surveillance in their respective countries [58]. The IgM sensitivity for ELISA and IFA assays were 87.8% (95% CI: 78.6–96.9).and 93.9% (95% CI: 85.8–100.0), respectively. For IgG assays, reported sensitivities were 80.4% (95% CI: 69.5–91.3) for ELISA and 86.1% (95% CI: 74.8–97.4) for IFA. The overall specificity was estimated at 100% for all the tests.

**Table 2:** Commercial serological assays for Crimean-Congo haemorrhagic fever virus detection as at 5 December 2017

Assay	Detection mode	Diagnostic kit producers	Comments/Target antigen
IgG ELISA		Vector-Best, Novosibirsk, Russia	Unknown
IgM ELISA	Qualitative		
IgG IFA test Ag detection			
IgG ELISA	Qualitative	Euroimmun, Luebeck, Germany	CCHFV GPC and CCHFV N
IgM ELISA			
IgG IFA			
IgG ELISA		Crimean-Congo ELISA Kits, Diagen Biyoteknolojik Sistemleri A.Ş., Ankara, Turkey	Unknown
IgM ELISA	Quantitative		
Ag ELISA			
IgG ELISA	Qualitative	Abbexa Ltd, Cambridge, United Kingdom	For research use only, not for diagnostic use.
IgM ELISA			
Elisa			
IgG ELISA	Quantitative	Alpha Diagnostic Intl. Inc., San Antonio, Texas, United States(US)	For research use only, not for diagnostic or therapeutic use. CCHFV NP
IgM ELISA	Quantitative		
IgA, IgG, IgM ELISA	Qualitative		
IgG ELISA	Qualitative	ELISA Kit, Antibody-Sunlong Biotech Co.,Ltd, Hangzhou, Zhejiang, China	Unknown
ELISA			
IgG ELISA	Qualitative	Creative Diagnostics, Shirley, New York, USA	CCHFV NP
IgM ELISA	Quantitative, qualitative		

CCHFV: Crimean-Congo haemorrhagic fever virus; ELISA: enzyme-linked immunosorbent assay; IFA: immunofluorescent assay; USA: United States of America.

A CCHFV seroneutralisation test is not normally performed for diagnostic purposes; it requires work with an infectious virus, necessitating a BSL4 laboratory, and is difficult to perform. However, reverse genetic approaches employing non-infectious reporter viruses have been described recently [59], enabling neutralisation to be performed at low containment.

### Virus isolation

Viral isolation, i.e. from blood or organ for further characterisation or infectivity studies, is performed under BSL4 conditions on either LLC-MK2, Vero, BHK-21 or SW-13.4 cell lines and can be achieved in 2–10 days [60]. CCHFV generally produces no or little cytopathogenic effect and viral growth can be detected by IFA with specific monoclonal antibodies [41] or by molecular tests. When viral isolation on cell cultures fails, it can be attempted in new-born or immunodeficient mice.

It is preferable that viral isolation is performed on samples collected during the first 5 days of infection, when the viraemia levels are high [58,61].

### Laboratory diagnosis of CCHFV infection

There is no official, agreed-upon case definition for CCHF in the EU, though several case definitions adopted by EU countries were reviewed by the European Centre for Disease Prevention and Control (ECDC) [62,63]. According to a report published by ECDC in 2014 [60], most countries used, at least for surveillance purposes, the EU case definition established for Viral Haemorrhagic Fever [64].

Box **Criteria proposed for laboratory confirmation of a clinically suspected Crimean Congo haemorrhagic fever case**

For laboratory confirmation of a clinical CCHF diagnosis, the expert group opinion is that a CCHFV infection is laboratory confirmed when at least one criteria in the Box is fulfilled.

• Detection by molecular tests of CCHFV RNA, in blood (whole blood, serum or plasma) or in other bodily fluids or tissues;

• Detection of CCHFV IgM or relevant (fourfold) increase in CCHFV IgG titres between two serologic samples (acute and convalescence phases);

• CCHFV isolation and/or detection of CCHF viral antigens in blood (whole blood, serum or plasma).

CCHFV: Crimean Congo haemorrhagic fever virus.

**Figure 3:**
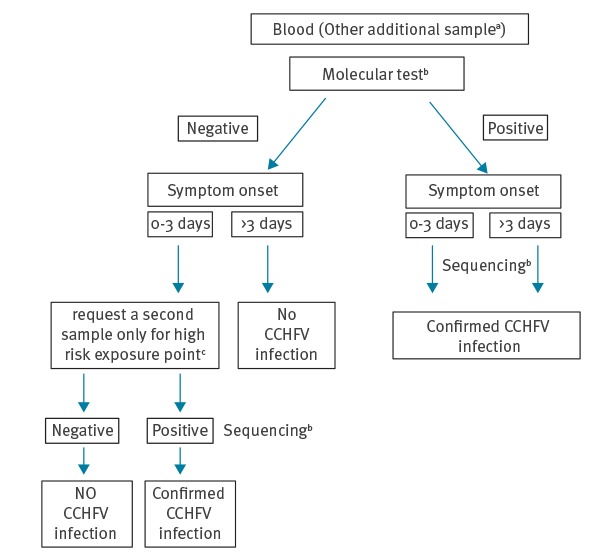
Algorithm for molecular diagnosis of Crimean-Congo haemorrhagic fever acute infection based on expert opinion

Taking into account that laboratory screening is usually performed using molecular methods, we propose as expert opinion the following molecular diagnostic algorithm for patients with suspected CCHF infection (Figure 3). However, it is advisable, especially when the molecular tests are negative, to perform also serological tests on all suspected patients.

CCHFV: Crimean-Congo haemorrhagic fever virus.

^a^ The preferred biological specimen is blood (serum, plasma or whole blood). Other possible biological specimens are saliva, urine [24,31] and post-mortem biopsy or bodily fluids (including semen, for infection control purposes [26,27]).

^b^ It is preferable that at least two different targets are tested: The first molecular test should target the S segment, while the second assay should be performed in a different genome region to confirm the absence or presence of CCHF infection, in case of negative result.

^c^ Sequencing is indicated for viral characterisation and cluster identification, as well as for the confirmation of first cases detected or of discordant results of molecular tests.

^d^ The molecular test to be performed should take into account epidemiological data.

Other relevant aspects related to management of CCHF patients as clinical manifestation and discharging criteria are reported in the Supplementary material.

## Discussion

CCHF is an important global health threat, as underlined by its inclusion in the list of priority diseases in the WHO document ‘An R & D blueprint for action to prevent epidemics’ [65].

In Europe, two autochthonous cases in Spain in 2016 [17] and the observed risk of importation of travel-associated cases [66] reinforce the notion that public health systems must be ready to respond to a potential emergence of CCHF.

Prompt and accurate laboratory diagnosis during the first days of the disease is critical to improve patient management, guide infection control measures and reduce case fatality. Early detection of viral RNA in blood is considered the gold standard diagnostic approach in the acute phase of the disease [21]. While the CCHFV RNA RT-PCR diagnostic test is most commonly performed in specialised laboratories, where non-commercial diagnostic assays and related reference biological material are available, this capability is more limited in rural areas and small cities where the majority of reported cases have occurred [9]. The availability of simple to use, commercial diagnostic tests will increase the number of laboratories performing RT-PCR or a similar NA detection strategy. However, laboratory capacity does not automatically mean capability, and laboratories implementing such tests would benefit from external quality assessments (EQA) of their capability to detect CCHFV in clinical samples, including monitoring of the effects of any corrective actions taken. The difficulty of clinical laboratories in establishing the diagnosis a CCHFV infection underlines the need to perform confirmatory tests in reference laboratories for both positive and negative samples collected from patients suspected of CCHF infection.

In addition, appropriate biosafety measures must be in place when performing molecular testing.

### Proposed measures to improve CCHFV laboratory preparedness and response

Measures to improve CCHFV laboratory preparedness and response should include: improving molecular tests to overcome the lack of sensitivity due to the high variability of the CCHFV genome; providing a positive control panel for molecular diagnostics, including the different CCHFV genotypes (including for serology testing) in order to support the improvement of diagnostic capability of clinical laboratories; enrolling diagnostic laboratories appointed for CCHFV diagnostics regularly in EQA programmes; improvement of diagnostic algorithms building on clinical experiences; validation of procedures to inactivate infectivity of clinical samples; and establishing of an international biorepository for the collection and storing of clinical samples, with the aim of validating new diagnostic tests and supporting pathogenicity studies. Some of these activities, including the evaluation of laboratory capability [67] and EQA [68] in particular, have been performed within the framework of EMERGE and EVD-LabNet and earlier as part of EU-funded projects such as EuronetP4 (Grant No. 2003214), EnP4Lab (Grant No. 2006208), QUANDHIP (Grant No. 20102102) and ENIVD (Framework Service Contract ref. no. ECDC/2008/011. Similar support of other collaborating EU projects, such as the European Virus Archive (EVAg), could meet these needs in the future.

## Conclusions

There are knowledge gaps concerning the putative persistence of the CCHFV in various body compartments of survivors and the related consequences for infection transmission. Basic knowledge is needed to provide evidence to better inform hospital discharge guidelines and these issues need further research. Furthermore, a One Health approach is required for adequate public health preparedness for CCHF, and relevant measures should include vector and animal surveillance, focusing particularly on migratory birds [5,9,69]. Greater awareness of the circulation of CCHFV in vectors/animals in specific geographic areas is fundamental in order to alert public health systems. Information on the circulation of CCHFV can be obtained by a syndromic approach and by including CCHFV testing in the diagnostic algorithm of severe febrile infectious diseases of unknown origin.

Until an effective vaccine and/or therapeutics have been developed, the CCHFV outbreak response will continue to rely on rapid identification and appropriate infection-control measures. Front-line hospitals, as well as reference laboratories, have a crucial role in the outbreak response, which should integrate laboratory, clinical and public health responses.

## Note

*This designation is without prejudice to positions on status, and is in line with United Nations Security Council Resolution 1244/99 and the International Court of Justice Opinion on the Kosovo Declaration of Independence.

## Supplementary Data

35000 Supplementary Material
