# Supplementary material for: Laboratory management of Crimean-Congo haemorrhagic fever virus infections: perspectives from two European networks
Source: Euro Surveill. 2019 Jan 31;24(5):1800093. doi: 10.2807/1560-7917.ES.2019.24.5.1800093 (PMC6386216; doi:10.2807/1560-7917.ES.2019.24.5.1800093)
Supplement: Supplementary Material [file 1800093_DiCARO_SupplementaryMaterial.docx]

Laboratory management of Crimean-Congo haemorrhagic fever virus infections: perspectives from two European networks

**SUPPLEMENT**

**Clinical manifestation of Crimean-Congo Hemorrhagic Fever (CCHF) and discharging criteria discharge criteria in patients with CCHF.**

This supplementary material is hosted by *Eurosurveillance* as supporting information alongside the article “Critical aspects of laboratory management of Crimean-Congo hemorrhagic fever virus infections: the point of view of two European networks dedicated to emerging diseases” on behalf of the authors who remain responsible for the accuracy and appropriateness of the content. The same standards for ethics, copyright, attributions and permissions as for the article apply. Eurosurveillance is not responsible for the maintenance of any links of email addresses provided therein.

**Clinical manifestation**

Clinical manifestation of CCHF varies from mild to severe [1] interestingly studies on seroprevalence in endemic areas suggest that a very high percentage of infection can be subclinical (up to 88%) [1,2]. However, the contribution that subclinical cases play in the transmission of the disease is unclear. The most common clinical symptoms are fatigue, fever, myalgia, headache, nausea and vomiting [2-4] while about 20% of patients have severe hemorrhagic manifestations (petechiae, epistaxis, ecchymosis, gingival bleeding, vaginal bleeding, bleeding in internal organs, cerebral and gastrointestinal bleeding) [3,5].

The incubation period is typically 1-7 days (range 1-13 days) days depending on the transmission route [5-8]: up to 7 days for transmission by tick bite and a median of 5 days after exposure to bodily fluids [9,10].

Hemorrhagic manifestations usually appear 3–6 days following onset of fever, in severe cases with a typical duration of 2-3 days [11, 12].

Viral load is a strong outcome predictor for CCHFV infection [13]. There is a significant correlation between the height of viral load and the fatality rate [14-18]. Other markers for fatal outcome are thrombocytopenia, increased clotting times, increased serum levels of ALT, AST, proinflammatory cytokines and chemokines, low CCHFV antibody titers and low platelet count [8, 10, 19].

The fatality rate, ranging from 3% to 70%, is highly variable and strictly dependent on the capability of the health system in the endemic area [11, 22, 21]. Bakir et al. analysing the mortality rate on the bases of a score system that takes into account different parameters (i.e. age, serum transaminase level, bleeding) estimated a mortality rate in high risk patients of up to 67% [22].

**Discharging criteria**

Discharging patients with viral hemorrhagic fever (VHFs) is challenging, due to concerns of transmitting the virus in the convalescence period [23]. In general, before discharging patients, preliminary steps to scale down isolation precautions to routine in-hospital practices should be applied.

In endemic countries, due to high laboratory overload and high bed occupancy, it is not feasible to test CCHFV RNA before discharge and the discharging practice of the tertiary care centres has been developed using clinical and not virological laboratory parameters [20]. Importantly the use of these clinical criteria alone has not met with complications, relapses, or secondary infections in a 30 day period following discharge [24].

However, in the absence of specific information the WHO recommend the following laboratory criteria of discharge that has been developed for other viral hemorrhagic diseases which have the potential for human-to-human transmission : a negative blood PCR on the third day of being asymptomatic or, if this PCR is positive, repeat test in 48 hours; if repeated PCR is negative, then patient can be discharged (WHO, 2016, <http://www.who.int/csr/resources/publications/clinical-management-patients/en/>, assessed on 21^st^ December 2017).

Convalescent patients and their contacts should be warned that some of the causative agents of VHF may continue to be excreted for many weeks in semen, as demonstrated with Marburg [25] and Ebola [25-27] viruses. Excretion of virus in urine and possibly faeces is also a risk. An anecdotic story of Lassa virus RNA persistence in urine up to 3 months after the diagnosis of the infection in an African patient co-infected by HIV, but with an unsuccessful attempt of virus isolation was been discussed several years ago at the meetings of BSL4 laboratory network (data not published), and persistence in semen 3 months after infection has been previously described (McCormick 2002 cited in Ergonul 2014). For CCHF, long lasting NA detection in blood and urine, up to 36 days from the onset of symptoms, have been described without evidence of infectivity [11] but no data are available on its persistence in semen and the possibility of sexual transmission is based only on epidemiological investigations [28,29].

The information of discharge criteria in patients with CCHF in Europe is limited.

For example, in Bulgaria, discharge is based mainly on clinical criteria and in some cases on lab PCR-neg results (Iva Christova, personal communication), while in Slovenia no specific discharge criteria are available (Tatjana Avsic, personal communication).

In Spain, the discharge criteria is the responsibility of the care services that attend to the patient and must be done individually. As a generic guideline, the patient is in an isolation unit (although asymptomatic) until viremia is negative. It is recommended that males who have recovered from CCHFV infection abstain from unprotected sexual intercourse (correct use of the condom) until semen has been confirmed negative by RT-PCR; if semen testing has not been carried out abstinence from unprotected sex for at least 6 months after the onset of symptoms is recommended. In the case of breastfeeding mothers, in the absence of evidence and precaution, it is recommended that convalescent females avoid breast feeding for a period of 15 days after recovery” (Maria Paz Sanchez-Seco, personal communication).

Due to the lack of more information and according to the precautionary criteria used by European countries during the recent Ebola outbreak, it is advisable to perform viral detection in bodily fluids (urine, cutaneous swabs) and discharge the patients only when PCR tests from the different samples are negative ( with the exception of semen). However, there are not real scientific evidences to support this advice and this diagnostic procedure probably cannot be performed in endemic countries with low resources, especially in an outbreak setting with high number of patients.

Concerning the possibility of sexual transmission, is advisable to perform molecular testing in semen and it might be recommended that the convalescent patient should adopt preventive measures (i.e. sexual abstinence or consistent condom use) for three months after clinical recovery [30,31]. Further researches addressing the real risk of CCHFV infection transmission by convalescent patients are needed in order to provide an evidence based discharge procedure.

**Bibliography**

1. Bodur H, E. Akinci, S. Ascioglu, P. Onguru, Y. Uyar Subclinical infections with Crimean-Congo hemorrhagic fever virus Turkey. Emerg. Infect. Dis., 18 (2012), pp. 640–642
2. Leblebicioglu H, Ozaras R, Irmak H, Sencan I. Crimean-Congo hemorrhagic fever in Turkey: Current status and future challenges. Antiviral Res. 2016; 126: 21–34. doi: 10.1016/j.antiviral.2015.12.003 PMID: 26695860
3. Yilmaz G.R., T. Buzgan, H. Irmak, A. Safran, R. Uzun, M.A. Cevik, M.A. Torunoglu. The epidemiology of Crimean-Congo hemorrhagic fever in Turkey, 2002-2007. Int J Infect Dis. 2009 May;13(3):380-6. doi: 10.1016/j.ijid.2008.07.021
4. Ozkurt Z, Kiki I, Erol S, Erdem F, Yilmaz N, Parlak M, Gundogdu M, Tasyaran MA Crimean-Congo hemorrhagic fever in Eastern Turkey: clinical features, risk factors and efficacy of ribavirin therapy. J Infect. 2006 Mar;52(3):207-15.
5. [Bente DA](https://www.ncbi.nlm.nih.gov/pubmed/?term=Bente%20DA%5BAuthor%5D&cauthor=true&cauthor_uid=23906741), Forrester NL, [Watts DM](https://www.ncbi.nlm.nih.gov/pubmed/?term=Watts%20DM%5BAuthor%5D&cauthor=true&cauthor_uid=23906741), [McAuley AJ](https://www.ncbi.nlm.nih.gov/pubmed/?term=McAuley%20AJ%5BAuthor%5D&cauthor=true&cauthor_uid=23906741), [Whitehouse CA](https://www.ncbi.nlm.nih.gov/pubmed/?term=Whitehouse%20CA%5BAuthor%5D&cauthor=true&cauthor_uid=23906741), [Bray M](https://www.ncbi.nlm.nih.gov/pubmed/?term=Bray%20M%5BAuthor%5D&cauthor=true&cauthor_uid=23906741). Crimean-Congo hemorrhagic fever: history, epidemiology, pathogenesis, clinical syndrome and genetic diversity. [Antiviral Res.](https://www.ncbi.nlm.nih.gov/pubmed/23906741) Oct;100(1):159-892013. doi: 10.1016/j.antiviral.2013.07.006.
6. Weidmann M, Avsic-Zupanc T, Bino S, Bouloy M, Burt F, Chinikar S, Christova I, Dedushaj I, El-Sanousi A, Elaldi N, Hewson R, Hufert FT, Humolli I, Jansen van Vuren P, Koçak Tufan Z, Korukluoglu G, Lyssen P, Mirazimi A, Neyts J, Niedrig M, Ozkul A, Papa A, Paweska J, Sall AA, Schmaljohn CS, Swanepoel R, Uyar Y, Weber F, Zeller H. Biosafety standards for working with Crimean-Congo hemorrhagic fever virus. J Gen Virol. 2016 Nov;97(11):2799-2808. doi: 10.1099/jgv.0.000610.
7. Nabeth P, Thior M, Faye O, Simon F. Human Crimean-Congo hemorrhagic fever, Senegal. Emerg Infect Dis. 2004 Oct;10(10):1881-2.
8. Ergonul O. Clinical and pathologic features of CrimeanCongo Hemorrhagic Fever. O Ergonul, CA Whitehouse, eds. Crimean-Congo Hemorrhagic Fever: A Global Perspective. First edition. 2007. Dordrecht: Springer, 207–220.
9. Naderi HR, Sheybani F, Bojdi A, Khosravi N, Mostafavi I. Fatal nosocomial spread of Crimean-Congo hemorrhagic fever with very short incubation period. Am J Trop Med Hyg. 2013 Mar;88(3):469-71.
10. Ergonul O, Celikbas A, Baykam N, Eren S, Dokuzoguz B. Analysis of risk-factors among patients with Crimean-Congo haemorrhagic fever virus infection: severity criteria revisited. Clin Microbiol Infect. 2006 Jun;12(6):551-4.
11. Thomas S, Thomson G, Dowall S, Bruce C, Cook N, Easterbrook L, O'Donoghue L, Summers S, Ajazaj L, Hewson R, Brooks T, Ahmeti S. Review of Crimean Congo hemorrhagic fever infection in Kosova in 2008 and 2009: prolonged viremias and virus detected in urine by PCR. Vector Borne Zoonotic Dis 2012;12:800–4.
12. Ergonul, O. Crimean-Congo haemorrhagic fever. Lancet Infect. Dis., 6 (2006), pp. 203-214
13. Papa A, Drosten C, Bino S, Papadimitriou E, Panning M, Velo E, Kota M, Harxhi A, Antoniadis A. Viral load and Crimean-Congo hemorrhagic fever. Emerg Infect Dis. 2007 May;13(5):805-6.
14. Duh, D Saksida, A., Petrovec, M., Ahmeti, S., Dedushaj, I., Panning, M., Drosten, C. & Avsic-Zupanc, T 2007. Viral load as predictor of Crimean-Congo hemorrhagic fever outcome. Emerg. Infect. Dis. 13, 1769e1772.
15. Saksida A, Duh D, Wraber B, Dedushaj I, Ahmeti S, Avsic-Zupanc T. Interacting roles of immune mechanisms and viral load in the pathogenesis of crimean-congo hemorrhagic fever. Clin. Vaccine Immunol. 17, 1086e1093; 2010
16. Kubar A., M. Haciomeroglu, A. Ozkul, U. Bagriacik, E. Akinci, K. Sener, et al., Prompt administration of Crimean-Congo hemorrhagic fever (CCHF) virus hyperimmunoglobulin in patients diagnosed with CCHF and viral load monitorization by reverse transcriptase-PCR, Jpn. J. Infect. Dis. 64 (2011) 439–443.
17. [Hasanoglu I](https://www.ncbi.nlm.nih.gov/pubmed/?term=Hasanoglu%20I%5BAuthor%5D&cauthor=true&cauthor_uid=26780111), Bakir M, Engin A, Kuskucu MA, Bakir S, Gündag O, Midilli K. Relationship of plasma cell-free DNA level with mortality and prognosis in patients with Crimean-Congo hemorrhagic fever. J Med Virol. 2016 Jul;88(7):1152-8. doi: 10.1002/jmv.24446.
18. Wölfel R, Paweska JT, Petersen N, Grobbelaar AA, Leman PA, Hewson R, Georges-Courbot MC, Papa A, Günther S, Drosten C. (2007) Virus detection and monitoring of viral load in Crimean-Congo hemorrhagic fever virus patients. Emerg Infect Dis 13: 1097–1100.
19. Papa A, Bino S, Velo E, Harxhi A, Kota M, Antoniadis A. Cytokine levels in Crimean-Congo hemorrhagic fever. J Clin Virol. 2006 Aug;36(4):272-6.
20. [Leblebicioglu H](https://www.ncbi.nlm.nih.gov/pubmed/?term=Leblebicioglu%20H%5BAuthor%5D&cauthor=true&cauthor_uid=26828701), [Sunbul M](https://www.ncbi.nlm.nih.gov/pubmed/?term=Sunbul%20M%5BAuthor%5D&cauthor=true&cauthor_uid=26828701), [Bodur H](https://www.ncbi.nlm.nih.gov/pubmed/?term=Bodur%20H%5BAuthor%5D&cauthor=true&cauthor_uid=26828701), [Ozaras R](https://www.ncbi.nlm.nih.gov/pubmed/?term=Ozaras%20R%5BAuthor%5D&cauthor=true&cauthor_uid=26828701); [Crimean–Congo Haemorrhagic Fever Research Network of Turkey (CCRNT)](https://www.ncbi.nlm.nih.gov/pubmed/?term=Crimean%E2%80%93Congo%20Haemorrhagic%20Fever%20Research%20Network%20of%20Turkey%20(CCRNT)%5BCorporate%20Author%5D). Discharge criteria for Crimean-Congo haemorrhagic fever in endemic areas. [J Infect.](https://www.ncbi.nlm.nih.gov/pubmed/?term=Discharge+criteria+for+CrimeaneCongo+haemorrhagic+fever+in+endemic+areas) 2016 Apr;72(4):500-1. doi: 10.1016/j.jinf.2016.01.009.
21. Papa A, Weber F, Hewson R, Weidmann M, Koksal I, Korukluoglu G, Mirazimi A. Meeting report: First International Conference on Crimean-Congo hemorrhagic fever. Antiviral Res. 2015 Aug;120:57-65. doi: 10.1016/j.antiviral.2015.05.005.
22. Bakir M, Engin A, Kuskucu MA, Bakir S, Gündag O, Midilli K. Relationship of plasma cell-free DNA level with mortality and prognosis in patients with Crimean-Congo hemorrhagic fever. J Med Virol. 2016 Jul;88(7):1152-8. doi: 10.1002/jmv.24446.
23. [Bevilacqua N](https://www.ncbi.nlm.nih.gov/pubmed/?term=Bevilacqua%20N%5BAuthor%5D&cauthor=true&cauthor_uid=26608193), [Nicastri E](https://www.ncbi.nlm.nih.gov/pubmed/?term=Nicastri%20E%5BAuthor%5D&cauthor=true&cauthor_uid=26608193), [Chinello P](https://www.ncbi.nlm.nih.gov/pubmed/?term=Chinello%20P%5BAuthor%5D&cauthor=true&cauthor_uid=26608193), [Puro V](https://www.ncbi.nlm.nih.gov/pubmed/?term=Puro%20V%5BAuthor%5D&cauthor=true&cauthor_uid=26608193), [Petrosillo N](https://www.ncbi.nlm.nih.gov/pubmed/?term=Petrosillo%20N%5BAuthor%5D&cauthor=true&cauthor_uid=26608193), [Di Caro A](https://www.ncbi.nlm.nih.gov/pubmed/?term=Di%20Caro%20A%5BAuthor%5D&cauthor=true&cauthor_uid=26608193), [Capobianchi MR](https://www.ncbi.nlm.nih.gov/pubmed/?term=Capobianchi%20MR%5BAuthor%5D&cauthor=true&cauthor_uid=26608193), [Lanini S](https://www.ncbi.nlm.nih.gov/pubmed/?term=Lanini%20S%5BAuthor%5D&cauthor=true&cauthor_uid=26608193), [Vairo F](https://www.ncbi.nlm.nih.gov/pubmed/?term=Vairo%20F%5BAuthor%5D&cauthor=true&cauthor_uid=26608193), [Pletschette M](https://www.ncbi.nlm.nih.gov/pubmed/?term=Pletschette%20M%5BAuthor%5D&cauthor=true&cauthor_uid=26608193), [Zumla A](https://www.ncbi.nlm.nih.gov/pubmed/?term=Zumla%20A%5BAuthor%5D&cauthor=true&cauthor_uid=26608193), [Ippolito G](https://www.ncbi.nlm.nih.gov/pubmed/?term=Ippolito%20G%5BAuthor%5D&cauthor=true&cauthor_uid=26608193); [INMI EbolaTeam](https://www.ncbi.nlm.nih.gov/pubmed/?term=INMI%20Ebola%20Team%5BCorporate%20Author%5D). Criteria for discharge of patients with Ebola virus diseases in high-income countries. [Lancet Glob Health.](https://www.ncbi.nlm.nih.gov/pubmed/26608193) 2015 Dec;3(12):e739-40.
24. [Leblebicioglu H](https://www.ncbi.nlm.nih.gov/pubmed/?term=Leblebicioglu%20H%5BAuthor%5D&cauthor=true&cauthor_uid=27424492), [Sunbul M](https://www.ncbi.nlm.nih.gov/pubmed/?term=Sunbul%20M%5BAuthor%5D&cauthor=true&cauthor_uid=27424492), [Barut S](https://www.ncbi.nlm.nih.gov/pubmed/?term=Barut%20S%5BAuthor%5D&cauthor=true&cauthor_uid=27424492), [Buyuktuna SA](https://www.ncbi.nlm.nih.gov/pubmed/?term=Buyuktuna%20SA%5BAuthor%5D&cauthor=true&cauthor_uid=27424492), [Ozkurt Z](https://www.ncbi.nlm.nih.gov/pubmed/?term=Ozkurt%20Z%5BAuthor%5D&cauthor=true&cauthor_uid=27424492), [Yapar D](https://www.ncbi.nlm.nih.gov/pubmed/?term=Yapar%20D%5BAuthor%5D&cauthor=true&cauthor_uid=27424492)6, [Yilmaz G](https://www.ncbi.nlm.nih.gov/pubmed/?term=Yilmaz%20G%5BAuthor%5D&cauthor=true&cauthor_uid=27424492), [Guner R](https://www.ncbi.nlm.nih.gov/pubmed/?term=Guner%20R%5BAuthor%5D&cauthor=true&cauthor_uid=27424492), [But A](https://www.ncbi.nlm.nih.gov/pubmed/?term=But%20A%5BAuthor%5D&cauthor=true&cauthor_uid=27424492), [Cicek Senturk G](https://www.ncbi.nlm.nih.gov/pubmed/?term=Cicek%20Senturk%20G%5BAuthor%5D&cauthor=true&cauthor_uid=27424492), [Murat N](https://www.ncbi.nlm.nih.gov/pubmed/?term=Murat%20N%5BAuthor%5D&cauthor=true&cauthor_uid=27424492), [Ozaras R](https://www.ncbi.nlm.nih.gov/pubmed/?term=Ozaras%20R%5BAuthor%5D&cauthor=true&cauthor_uid=27424492), [Crimean Congo Hemorrhagic Fever Research Network of Turkey](https://www.ncbi.nlm.nih.gov/pubmed/?term=Crimean%20Congo%20Hemorrhagic%20Fever%20Research%20Network%20of%20Turkey%5BCorporate%20Author%5D). Multi-center prospective evaluation of discharge criteria for hospitalized patients with Crimean-CongoHemorrhagic Fever. [Antiviral Res.](https://www.ncbi.nlm.nih.gov/pubmed/?term=Multi-center+prospective+evaluation+of+discharge+criteria+for+hospitalized+patients+with+Crimean-Congo+Hemorrhagic+Fever) 2016 Sep;133:9-13. doi: 10.1016/j.antiviral.2016.07.010.
25. Brainard J, Pond K, Hooper L, Edmunds K, Hunter P. Presence and Persistence of Ebola or Marburg Virus in Patients and Survivors: A Rapid Systematic Review. PLoS Negl Trop Dis. 2016 Feb 29;10(2):e0004475. doi: 10.1371/journal.pntd.0004475. eCollection 2016 Feb. Review.
26. Sissoko D, Duraffour S, Kerber R, Kolie JS, Beavogui AH, Camara AM, Colin G, Rieger T, Oestereich L, Pályi B, Wurr S, Guedj J, Nguyen TH, Eggo RM, Watson CH, Edmunds WJ, Bore JA, Koundouno FR, Cabeza-Cabrerizo M, Carter LL, Kafetzopoulou LE, Kuisma E, Michel J, Patrono LV, Rickett NY, Singethan K, Rudolf M, Lander A, Pallasch E, Bockholt S, Rodríguez E, Di Caro A, Wölfel R, Gabriel M, Gurry C, Formenty P, Keïta S, Malvy D, Carroll MW, Anglaret X, Günther S. Persistence and clearance of Ebola virus RNA from seminal fluid of Ebola virus disease survivors: a longitudinal analysis and modelling study. Lancet Glob Health. 2017 Jan;5(1):e80-e88. doi: 10.1016/S2214-109X(16)30243-1.
27. Smith JR, Todd S, Ashander LM, Charitou T, Ma Y, Yeh S, Crozier I, Michael MZ, Appukuttan B, Williams KA, Lynn DJ, Marsh GA. Retinal Pigment Epithelial Cells are a Potential Reservoir for Ebola Virus in the Human Eye. Transl Vis Sci Technol. 2017 Jul 14;6(4):12. doi: 10.1167/tvst.6.4.12.
28. Ergonul O, Battal I. Potential sexual transmission of Crimean-Congo hemorrhagic fever infection. Jpn J Infect Dis 2014;67:137–8.
29. Pshenichnaya NY, Sydenko IS, Klinovaya EP, Romanova EB, Zhuravlev AS. Possible sexual transmission of Crimean-Congo hemorrhagic fever. Int J Infect Dis. 2016 Apr;45:109-11. doi: 10.1016/j.ijid.2016.02.1008.
30. Borio L, Inglesby T, Peters CJ, Schmaljohn AL, Hughes JM, Jahrling PB, Ksiazek T, Johnson KM, Meyerhoff A, O'Toole T, Ascher MS, Bartlett J, Breman JG, Eitzen EM Jr, Hamburg M, Hauer J, Henderson DA, Johnson RT, Kwik G, Layton M, Lillibridge S, Nabel GJ, Osterholm MT, Perl TM, Russell P, Tonat K; Working Group on Civilian Biodefense. Hemorrhagic fever viruses as biological weapons: medical and public health management. JAMA, 287 (2002), pp. 2391-2405
31. Ergonul O. Crimean-Congo hemorrhagic fever virus: new outbreaks, new discoveries. Curr Opin Virol. 2012 Apr;2(2):215-20. doi: 10.1016/j.coviro.2012.03.001.
